# DNA and RNA Methylation in Periodontal and Peri-implant Diseases

**DOI:** 10.1177/00220345241291533

**Published:** 2024-12-04

**Authors:** L. Larsson, P.M. Giraldo-Osorno, C. Garaicoa-Pazmino, W.V. Giannobile, F. Asa’ad

**Affiliations:** 1 Department of Oral Biochemistry, Institute of Odontology, Sahlgrenska Academy, University of Gothenburg, Sweden; 2 Department of Biomaterials, Institute of Clinical Sciences, Sahlgrenska Academy, University of Gothenburg, Sweden; 3 Department of Periodontics, University of Iowa College of Dentistry, Iowa City, Iowa, USA; 4 Research Center, School of Dentistry, Universidad de Especialidades Espiritu Santo, Samborondón, Ecuador; 5 Department of Oral Medicine, Infection, and Immunity, Harvard School of Dental Medicine, Boston, MA, USA

**Keywords:** epigenetic(s), periodontal disease(s)/periodontitis, peri-implant infection(s), molecular biology, inflammation, methylgroup

## Abstract

Periodontal and peri-implant diseases are primarily biofilm-induced pathologies in susceptible hosts affecting the periodontium and dental implants. Differences in disease susceptibility, severity, and patterns of progression have been attributed to immune regulatory mechanisms such as epigenetics. DNA methylation is an essential epigenetic mechanism governing gene expression that plays pivotal roles in genomic imprinting, chromosomal stability, apoptosis, and aging. Clinical studies have explored DNA methylation inhibitors for cancer treatment and predictive methylation profiles for disease progression. In periodontal health, DNA methylation has emerged as critical, evidenced by clinical studies unraveling its complex interplay with inflammatory genes and its regulatory role in periodontitis contributing to disease severity. Human studies have shown that methylation enzymes associated with gene reactivation (e.g., ten-eleven translocation-2) are elevated in periodontitis compared with gingivitis. Dysregulation of these genes can lead to the production of inflammatory cytokines and an altered initial response to bacteria via the toll-like receptor signaling pathway in periodontal diseases. In addition, in peri-implant diseases, this dysregulation can result in altered DNA methylation levels and enzymatic activity influenced by the properties of the titanium surface. Beyond traditional perspectives, recent evidence highlights the involvement of RNA methylation (e.g., N6-methyladenosine [m6A], N6,2′-0-dimethyladenosine [m6Am]) in periodontitis and peri-implantitis lesions, playing vital roles in the innate immune response, production of inflammatory cytokines, and activation of dendritic cells. Both DNA and RNA methylation can influence the gene expression, virulence, and bacterial behavior of well-known periodontal pathogens such as *Porphyromonas gingivalis*. Alterations in bacterial methylation patterns result in changes in the metabolism, drug resistance, and gene expression related to survival in the host, thereby promoting tissue degradation and chronic inflammatory responses. In summary, the present state-of-the-art review navigates the evolving landscape of DNA and RNA methylation in periodontal and peri-implant diseases, integrating recent developments and mechanisms to reshape the understanding of epigenetic dynamics in oral health.

## Introduction

**Figure 1.**
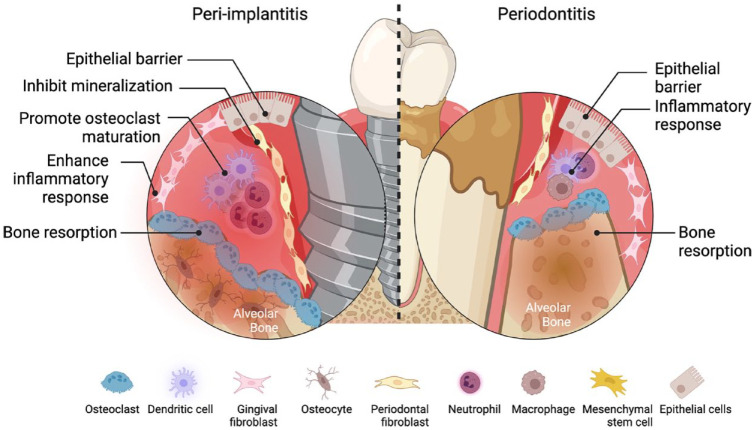
Comparison of periodontitis and peri-implantitis showing the major cell types present in the inflammatory lesions.

Distinct microbial and histomorphometric features differentiate gingivitis, periodontitis, and peri-implantitis beyond traditional clinical and radiographic parameters as observed in naturally occurring and experimental human studies. Periodontitis lesions are twice as large as biofilm-induced gingivitis lesions, containing a higher proportion of plasma cells and macrophages (Thorbert-Mros et al. 2015), whereas peri-implantitis lesions are double in size compared with periodontitis lesions and thus contribute to dissimilarities in onset and progression between these diseases (Carcuac and Berglundh 2014) (Fig. 1). Although disease susceptibility, bacterial colonization, and the host response are critical factors in the development of periodontal and peri-implant diseases, several immune regulatory mechanisms have been identified as possibly modulating the onset and disease progression of these conditions.

**Figure 2.**
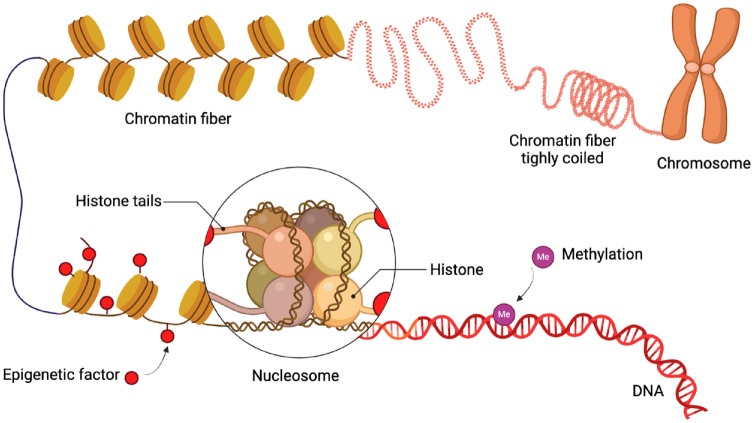
Schematic overview of epigenetic modifications of chromatin condensation. Me, methylation. Red circles indicate histone acetylation.

Epigenetics refer to the chemical alterations in gene expression not encoded in the DNA sequence leading to changes in the remodeling of the chromatin structure (Bird 2002) (Fig. 2). Epigenetic modifications (e.g., histone acetylation, DNA methylation, RNA methylation) are reversible and dynamic in response to several factors including the oral microbiome, environmental habits (e.g., smoking), age, dietary compounds, and so forth. These regulatory mechanisms may provide explanations as to why patients with the same clinical phenotype display biological processes (e.g., inflammation) and disease susceptibility and/or different responses to treatment. In addition, epigenetic changes are cell and tissue specific, an important factor in chronic inflammatory diseases such as periodontal and peri-implant diseases.

Both cellular composition and the oral microbiome contribute to the epigenetic pattern of a tissue/disease. For example, an upregulation of the RNA methylation levels in macrophages has been shown to influence their polarization into a M1 bactericidal phenotype (Sun, Ren, et al. 2024). N6-methyladenosine (m6A) is RNA methylation modification present in bacteria and can affect host cells, and vice versa, the host microbiome can affect bacteria RNA methylation. Thus, m6A has been suggested to mediate crosstalk between the host and microbiome in the gut (Zhuo et al. 2022). At present, there is a lack of knowledge about the mechanisms associated with the oral microbiome as well. With regard to DNA methylation, the release of titanium particles/ions around dental implants could contribute to peri-implantitis through various mechanisms, including DNA methylation alterations and shaping the oral microbiome (Asa’ad et al. 2022). The presence of titanium particles can modulate lymphocyte and macrophage polarization in peri-implant gingival tissues, further influencing the immune response in the peri-implant environment (Kheder et al. 2023).

Over the past decade, DNA methylation has been extensively explored, whereas RNA methylation has gained emerging interest in recent years. The present state-of-the-art review will focus on DNA and RNA methylation modifications and their role in periodontal and peri-implant diseases.

## Regulation of the Immune Response via DNA Methylation

DNA can be modified by the addition of methyl groups to cytosine bases situated next to a guanine base at specific sites in the DNA sequence (i.e., CpG islands). This modification is referred to as 5-methyl cytosine (5mC) and leads to the inactivation of gene expression (Bird 2002). 5mC can be further oxidized into 5-hydroxymethylcytosine (5hmC) by the ten-eleven translocation (TET) family of enzymes (Tahiliani et al. 2009). This oxidation has been suggested as the mechanism for the demethylation of DNA so that the cell can reactivate genes (Kraus et al. 2012).

DNA methylation is a major epigenetic mechanism that involves the methylation of the 5mC within DNA, particularly at the CpG sites, thereby inducing gene silencing. This process is primarily orchestrated by DNA methyltransferase (DNMT) enzymes. These enzymes are categorized into 2 groups: de novo methylation enzymes (e.g., DNMT3A and DNMT3B) responsible for initiating methylation and methylation maintenance enzymes (e.g., DNMT1), which ensures the preservation of methylation patterns during replication (Hervouet et al. 2018).

Conversely, DNA demethylation occurs through 2 distinct pathways: passive and active. Passive demethylation is initiated by the inhibition or absence of DNMT1, thus hindering the methylation process. In contrast, active DNA demethylation is regulated by TET, a well-known DNA hydroxymethylation enzyme (Wu and Zhang 2010). TETs facilitate the oxidation of 5mC to 5hmC, initiating the process of hydroxymethylation. Subsequent oxidation steps lead to the formation of 5-formylcytosine and then 5-carboxycytosine, which is subsequently repaired by glycosylase through the base excision repair pathway of DNA (Shen et al. 2014). Thus, TETs play a crucial role in promoting gene expression through DNA demethylation mechanisms.

### DNA Methylation and Periodontal Disease

Oral pathogens and their products, such as lipopolysaccharides (LPS), have been implicated in the induction of methylation alterations during the gene expression of host cells and tissues, thus influencing periodontitis (Appendix Table 1). The influence of *Treponema denticola* on periodontal ligament stem cells (PDLSCs) has been investigated, and even though a hypomethylation of the matrix metalloprotease-2 (MMP-2) promoter was found, the low levels may not have any biological influence on the gene expression of MMP-2 (Miao et al. 2014). Furthermore, it has been demonstrated that *Porphyromonas gingivalis* and *Fusobacterium nucleatum* downregulate the expression of DNMT1 in oral epithelial cells (Martins et al. 2016).

Increased DNA methylation of the toll-like receptor-2 (TLR-2) promoter in gingival epithelial cells cultured with *P. gingivalis* has been demonstrated, highlighting the role of the DNA methylation in modulating immune responses (Yin and Chung 2011). Dysregulation of TLR expression and subsequent alterations in the host response against periodontal pathogens may exacerbate inflammation and increase susceptibility to periodontitis (Benakanakere et al. 2015). In a study by De Oliveira et al. (2011), it was observed that the promoter region of TLR-4 remained unmethylated in both healthy individuals and those diagnosed with periodontitis. Conversely, the promoter region of TLR-2 exhibited a combination of methylated and unmethylated regions in both cohorts. Interestingly, a significantly elevated degree of TLR-2 promoter methylation was detected in periodontitis patients compared with the healthy group in another study (de Faria Amormino et al. 2013).

Different studies have also explored DNA methylation patterns of inflammatory cytokines and markers, revealing substantial differences between healthy and periodontitis patients (De Souza et al. 2014; Schulz et al. 2016). In fact, variations in DNA methylation levels have been observed in genes related to immune responses against bacteria, including interleukin-17C (IL-17C) and chemokine ligand-25 (Schulz et al. 2016). A recent study has shown that generalized periodontitis is associated with hypomethylation of the signal transducer and activator of the transcription 5 (STAT5) gene, which plays a role in the production of different cytokines (Azevedo et al. 2020). Interestingly, a cross-sectional study demonstrated that the DNA methylation levels of the genes of the inflammatory cytokines tumor necrosis factor alpha (TNF-α) and interferon gamma (IFN-γ) were sustained despite periodontal therapy (Asa’ad et al. 2017).

An epigenome-wide association study investigated the association between CpG-specific DNA methylation in peripheral blood samples and periodontal disease, identifying genomic regions linked to periodontitis and edentulism (Zhao et al. 2023). Differential DNA methylation was identified in specific regions of 2 transcription factors: hypomethylation in Zinc finger protein-57 (ZFP57) and hypermethylation in homeobox A4 (HOXA4) genes.

Despite extensive research on DNA methylation alterations associated with periodontitis, few studies have investigated the expression of DNA methylation and hydroxymethylation markers themselves. Martins et al. (2016) reported a downregulation of DNMT1 in oral epithelial cells near inflammatory lesions in a ligature-induced periodontitis model. Moreover, tissue samples from periodontitis patients have shown an upregulation of DNMT1 and TET1 messenger RNA (mRNA) compared with healthy controls, indicating dysregulated epigenetic processes in diseased tissues (De Souza et al. 2014). In addition, Larsson et al. (2016) reported an increase in TET2-positive cells in periodontitis lesions relative to gingivitis lesions, thus suggesting a link between disease severity and the epigenetic regulation of inflammatory genes.

The relationship between genetic polymorphisms and DNA methylation and hydroxymethylation markers has been recently explored in periodontitis. Asa’ad and coworkers (2023) demonstrated that homozygous carriage of the minor A-allele of DNMT1 was associated with decreased susceptibility to periodontitis and that the minor A-allele of TET2 was associated with increased susceptibility to periodontitis, whereas Coêlho et al. (2020) concluded that the T allele and the TT genotype of DNMT3B were detected more frequently in the periodontitis group compared with healthy individuals.

### DNA Methylation and Peri-implantitis

To date, there are 2 clinical studies available on DNA methylation and peri-implantitis (Daubert et al. 2019; Khouly et al. 2022) (Appendix Table 1). In a cross-sectional study, the assessment of global DNA methylation in soft tissues and bone retrieved from failed dental implants revealed comparable global DNA methylation levels between peri-implant health and peri-implantitis–affected implants (Khouly et al. 2022). Notably, a distinctive feature was identified, as the soft tissues exhibited higher DNA methylation levels compared with the alveolar bone, thus highlighting the specificity of epigenetic alterations within distinct microenvironments. Unfortunately, the study did not provide sufficient information of numerous factors, precluding the establishment of a direct link between surface characteristics and pathogenic processes.

Similarly, global DNA methylation and quantified titanium particles were explored within the peri-implant crevicular fluid (PICF) derived from both peri-implantitis–affected and healthy implants (Daubert et al. 2019). Their findings demonstrated an elevated global DNA methylation level among peri-implantitis. Interestingly, the increased global DNA methylation levels coincided with heightened titanium quantities in PICF, irrespective of the peri-implant status. However, the lack of information on implant surfaces in this study also prevented the extrapolation of any direct association between implant surface characteristics and global DNA methylation patterns.

## Regulation of the Immune Response via RNA Methylation

**Figure 3.**
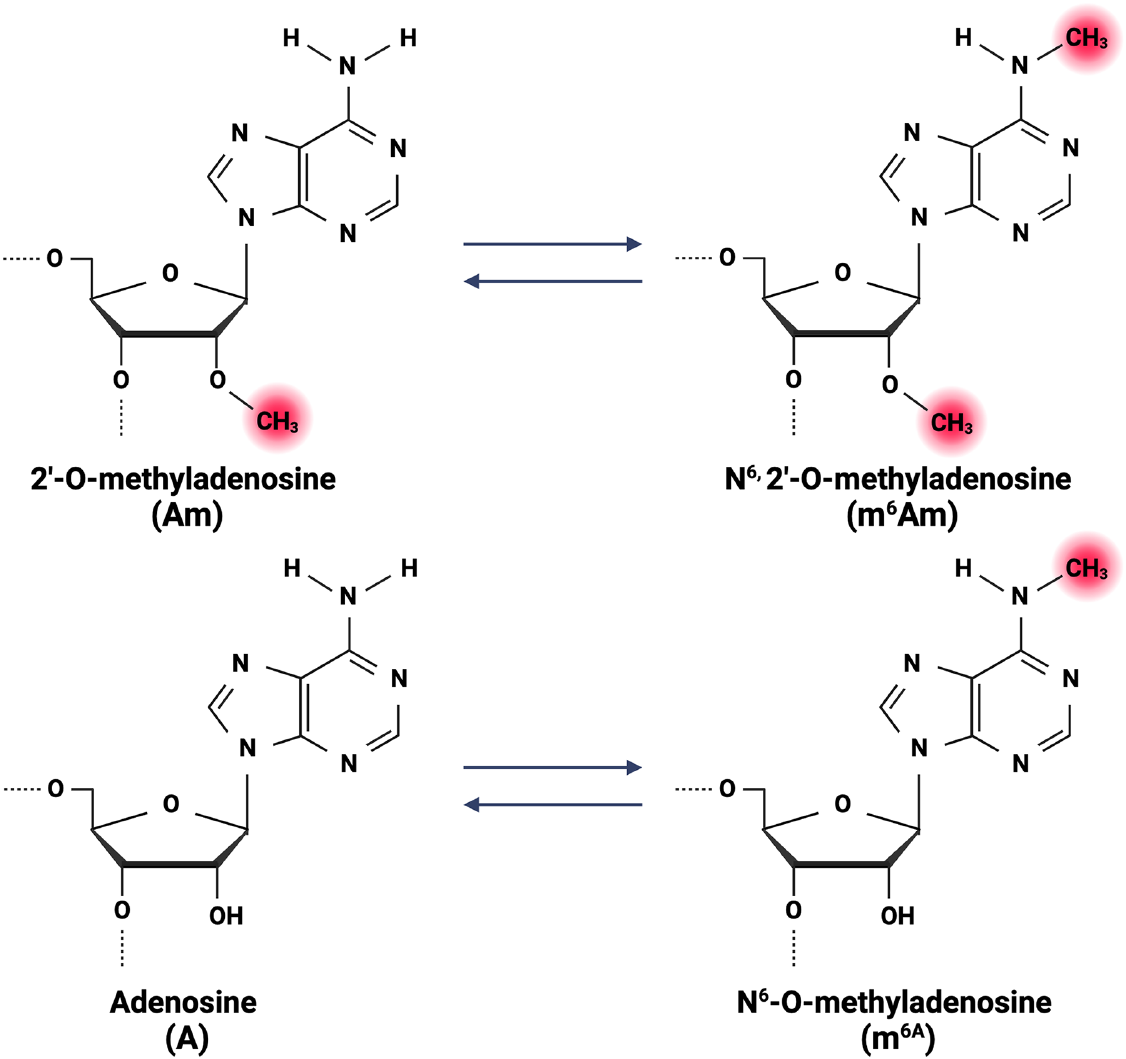
Illustration of the various RNA methylation forms.

Numerous RNA modifications have been identified including m6A, N6,2′-0-dimethyladenosine (m6Am), N6-hydroxymethyladenosine (hm6A), and 5′ cap structure N7-methylguanosine (m7G) (Oerum et al. 2021) (Fig. 3). These modifications contribute not only to the regulation of gene expression but also to the development of diseases (e.g., chronic inflammatory diseases and cancer). Among these, m6A is the most common RNA modification sharing ties with m6Am. Therefore, the review will focus on this RNA modification.

The m6A modification is considered a posttranscriptional marker present in several RNAs including mRNA, transfer RNA (tRNA), ribosomal RNA (rRNA), and micro-RNA (miRNA), and it has been found to regulate RNA splicing, translation, stability, and translocation (Dominissini et al. 2012; Jiang et al. 2021). Although the m6A modification was identified in the mid-1970s, only modern sequencing techniques have made it possible to study its mechanisms and functions at a cellular level.

Emerging evidence indicates that m6A and associated proteins play a vital role in the innate immune response by recognizing and regulating the response to pathogens (e.g., bacteria and viruses), production of inflammatory cytokines, and activation of dendritic cells (Tang et al. 2021). Moreover, the differentiation and function of T cells and B cells have also been found to be regulated by m6A and to play a role in the adaptive immune response. Dysregulation of m6A and its related proteins has been reported in several immune-related diseases (e.g., multiple sclerosis, rheumatoid arthritis, among others) (Tang et al. 2021).

m6A are regulated by “writers” (i.e., methylases) that add a methyl group, “erasers” (i.e., demethylases) responsible for removing the methyl group, and “readers” that can either mediate, promote, or enhance splicing, translation, degradation, and stability of RNA via m6A recognition (Dominissini et al. 2012; Jiang et al. 2021) (Appendix Table 2). The same reader can have opposite functions depending on the cell type and physiological and pathological conditions.

Methyltransferase-like (METTL) (e.g., METTL3, METTL13, METTL14, METTL16) and Vir-like m6A-associated (VIRMA) proteins are among the most common writers. METTL3 is the most extensively studied METTL capable of methylating genes involved in several processes, such as cell reprogramming and T-cell homeostasis (Jiang et al. 2021). Wilms tumor 1-associated protein (WTAP) is a regulatory component of m6A methyltransferases that stabilizes METTL3 and METTL4 by forming a catalytic core that will install m6A into an RNA target (Tang et al. 2021; Lan et al. 2022).

**Figure 4.**
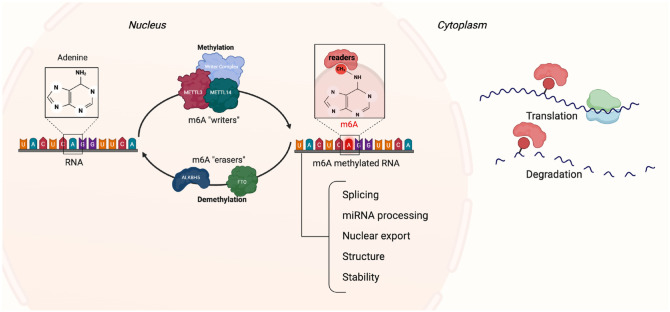
Schematic drawing of the process of RNA m6A modification. m6A modification is regulated by several m6A methyltransferases (writers) and demethylases (erasers). Readers in the nucleus and cytoplasm interact with the m6A, resulting in regulation of various processes. m6A, N6-methyladenosine.

On the other hand, fat mass and obesity-associated protein (FTO) and Alkylation B homolog 5 (ALKBH5) are the 2 most common erasers (Jiang et al. 2021; Tang et al. 2021). FTO was the first demethylase for m6A to be identified (Jia et al. 2011), and it was shown that an increase in FTO resulted in a decrease in m6A. Similar to DNA demethylases, FTO oxidizes m6A into hm6A, which is then further oxidized by FTO into N6-formyladenosine (f6A) and finally into adenine (Huang, Guo, Liu, et al. 2023). Moreover, ALKBH5 plays a role in the inflammatory response by regulating the m6A level of genes related to T-cell regulation and the accumulation and migration of neutrophils to the site of inflammation and macrophage recruitment. Thus, it plays a vital role in the resolution of inflammation (Liu, Song, et al. 2022). This shows that m6A, like DNA methylation, is a reversible process (Fig. 4).

Readers can be divided into 2 groups depending on their location. The first group is the nuclear readers, which include YTH domain containing 1-2 (YTHDC1-2), heterogeneous nuclear ribonucleoproteins A2/B1 (HNRNPA2B1), HNRNPC, HNRNPG, and fragile X messenger ribonucleoproteins (FMRs). Nuclear readers are involved in several biological functions such as mRNA splicing, nuclear export of mRNA, and regulation of noncoding RNAs (Tang et al. 2021). The second group is the cytoplasmic readers and includes YTHDC2, YTHDF1-3, insulin-like growth factor-binding protein-2 (IGF2BPs), FMRs, and proline-rich coiled-coil2A (Prrc2a). These readers regulate mRNA stability, translation, and mRNA degradation (Tang et al. 2021; Liu, Zheng, et al. 2022). The m6A reader YTHDC1 can promote exon inclusion and facilitate nuclear transportation of m6A methylated transcripts, while the readers YTHDF1-3 and YTHDC2 enhance translation. YTHDF3 can together with YTHDF2 work synergizing to promote protein synthesis (Jiang et al. 2021).

Ultimately, there is limited evidence about m6Am mostly because there have not been efficient methods to identify and analyze it. Recent reports on m6Am have found that this RNA modification is located adjacent to the m7G cap on mRNA, and it is only when the first transcribed nucleotide is adenosine that it can be modified into m6Am (Sun et al. 2021; Cesaro et al. 2023).

The readers for m6Am are METTL4 and phosphorylated CTD-interacting factor 1 (PCIF1) also known as cap-specific adenosine methyltransferase (CAPAM) (Sun et al. 2021; Cesaro et al. 2023). The function of m6Am remains inconclusive, with conflicted findings on its role in RNA stability and translation (Cesaro et al. 2023). Although there is still limited knowledge about the role of m6Am in disease, it has been shown to be regulated by heat shock and hypoxia, suggesting a potential role in cellular stress response (Sun et al. 2021). Emerging reports also indicate a role for m6Am in tumorigenesis (Cesaro et al. 2023). Both PCIF1 and FTO have been found to be reduced in metastatic cancer cell lines, with FTO expression particularly low in patient-derived colon cancer cell lines. This reduction leads to an increase in m6Am, which enhances tumorigenesis and chemoresistance (Relier et al. 2021).

### RNA Methylation and Periodontal Disease

Using biopsies from periodontitis lesions and healthy tissues, 23 m6A regulators were analyzed, and 17 were found to be dysregulated between health and disease with YTHCD1, WTAP, and ALKBH5 as the most important regulators (Zhang et al. 2021). Interestingly, correlating the regulators with inflammatory markers resulted in 3 subtypes of periodontitis, each with their unique immune characteristics and 2 of those having more infiltrating immune cells (Zhang et al. 2021). Another study, also using a set of databases, found 104 m6A-associated single-nucleotide polymorphisms (m6A-SNPs) to be associated with periodontal disease, and the rs2723186 m6A-SNP was identified as potentially regulating IL-37 gene expression in periodontal disease (Lin et al. 2020).

Saliva samples from periodontitis patients were analyzed for free nucleoside composition and the composition of nucleosides from RNA (Vignon et al. 2023). It was concluded that among free nucleosides, inosine, queuosine, and m6Am were altered in periodontitis patients. Furthermore, m6Am was significantly lower among patients with a moderate disease progression (grade B), and nucleosides from digested RNA uridine were significantly higher in severe forms of periodontitis (stage 3) and Grade B groups compared with healthy individuals (Vignon et al. 2023). Based on these results, it was suggested that m6Am could be a potential biomarker for periodontitis.

The knockdown of METTL3, as well as METTL14, can lead to a decrease in the osteogenic markers alkaline phosphatase (ALP), alpha-1 type I collagen (COL1A1), and runt-related transcription factor-2 (RUNX2) in PDLSCs (Huang, Guo, Wang, et al. 2023). Similarly, Sun, Meng, et al. (2024) reported an increase in METTL3 and m6A levels as well as RUNX2 expression during osteogenic differentiation in PDLSCs. Overexpression of METTL3 resulted in upregulation of RUNX2, and knockdown of METTL3 inhibited osteogenic differentiation and reduced expression of RUNX2 (Sun, Meng, et al. 2024). Inhibition of METTL3 in vivo resulted in alveolar bone and collagen destruction, an increase in osteoblasts, as well as an increase in inflammatory cell infiltrate (Zhang, Kong, et al. 2024).

In addition, changes in m6A pattern influencing the stability of long noncoding RNAs (lncRNAs) have been suggested as a mechanism influencing the osteogenic potential of PDLSCs (Sun et al. 2022; Zhang, Liu, et al. 2024). PDLSCs from periodontitis patients exposed to static mechanical stress had a different m6A pattern and a decrease in METTL3 compared with cells from healthy individuals (Sun et al. 2022). Chen et al. (2024) found that METTL3 promoted osteogenic differentiation of PDLSCs from periodontitis patients by regulating the stability and expression of the lncRNA cutA divalent cation tolerance homolog-like protein (CUTALP) that in turn regulated the microRNA-RUNX2 pathway.

METTL3 and METTL14 not only influence the osteogenic potential of PDLSCs, but they can also influence the inflammatory response in periodontitis. LPS stimulation of PDLSCs increased the total m6A levels and expression of METTL3 and METTL14 (Huang et al. 2024). Interestingly, knockdown of METTL3 and METTL14 suppressed LPS-induced expression of IL-6.

Insulin-like growth factor 2 mRNA-binding protein-1 (IGF2BP1) upregulation was observed during osteogenic differentiation, whereas overexpression can occur when combined with an increase of RUNX2 mRNA and protein expression. As METTL3 was downregulated, the IGF2BP1-mediated stability of RUNX2 mRNA was reduced. Therefore, it was suggested that the osteogenic differentiation of PDLSCs was a result of IGF2BP1-mediated RUNX2 mRNA stability (Sun, Meng, et al. 2024).

IGF2BP2 expression was also found in high levels in alveolar bone, periodontal ligament, and gingival tissue from patients with periodontitis. Using a ligature-induced periodontitis murine model increased the level of IGF2BP2 found at 3 d in alveolar bone, and the expression decreased after 14 d (Ma et al. 2024). The same trend was found for IL-1β, IL-6, and TNF-α, thereby suggesting a role for IGF2BP2 in the regulation of inflammation in periodontitis.

Tissue samples from periodontitis patients and healthy controls were analyzed using real-time polymerase chain reaction, m6A immunoprecipitation, and microarray (Wang et al. 2023). The results showed a different m6A pattern in periodontal and healthy tissues and verified 2 downregulated biomarkers and genes (delta and notch-like epidermal growth factor-related receptor [DNER] and nucleolar guanosine triphosphate-binding protein-2 [GNL2]), which may provide novel insights into new molecular mechanisms and latent targets of periodontitis. DNER is a transmembrane protein that has been linked to IFN-γ expression in chronic inflammation. Moreover, GNL2 is involved in protein synthesis, cell-cycle regulation, and nucleolar signaling.

The accumulation of advanced glycation end products (AGEs) has been suggested to contribute to type 2 diabetes as well as to periodontitis by influencing cellular functions and contributing to inflammation. Using an in vivo model, bone marrow stem cells (BMSCs) treated with AGEs induced inhibition of ALP activity, an increase in m6A and FTO levels, and a decrease in METTL3, METTL14, and ALKBH5 (Zhou et al. 2023). It was suggested that FTO may have a role in AGE impairment of BMSC differentiation into osteoblasts and influence FTO expression and m6A levels.

METTL3 is not the only m6A-related marker that has been found to contribute to periodontal disease. In an experimental apical periodontitis model, FTO expression was suppressed in cementoblasts, whereas FTO knockdown upregulated the inflammatory response (e.g., IL-6, chemokine C-C motif ligand 2 [CCL2], matrix metalloproteinase-9 [MMP9], activation of nuclear factor kappa-light-chain-enhancer of activated B cells [NFκB], and mitogen-activated protein kinase [MAPK] signaling pathways) but not affecting the mRNA stability of inflammation-related genes (Li et al. 2023). Appendix Table 3 depicts a summary of the main findings and characteristics of studies evaluating RNA modifications in periodontal models.

### RNA Methylation and Peri-implant Diseases

To date, there has been only a single published investigation on m6A methylation in peri-implantitis (Krishnamoorthy et al. 2023) (Appendix Table 3). In that study, soft-tissue biopsies were harvested to investigate the role of m6A modification and METTL3 among patients with peri-implantitis. There were significantly high levels of m6A and elevated gene/protein expression of METTL3 in peri-implantitis when compared with peri-implant health. Thus, it was suggested that dysregulation of m6A modification is associated with peri-implantitis and is possibly a strong risk factor for peri-implantitis.

## Clinical Implications and Future Directions

As in cancer research, drug-assisted therapies targeting epigenetic modifications have been considered to control disease activity of periodontal and peri-implant diseases. Epigenetic inhibitors (e.g., histone deacetylases, histone acetyltransferases, and DNMTs) could represent novel and potential agents that can be used as adjuncts to active/supportive therapy to induce repair of any cellular damage caused by oral inflammation (Larsson et al. 2015). Furthermore, pharmacoepigenetic biomarkers may be used to assess drug effectiveness and predict the response of periodontal and peri-implant therapy.

Targeting the m6A modification has been suggested in tumor immunity and viral detection/response, and it has been proposed to have a potential contributory role in autoimmune diseases (Tang et al. 2021). Potential drugs such as CS1, CS2, and NSC48890, suggested for acute myeloid leukemia, and imidazobenzoxazin-5-thione (MV1035) aiming for tumor immunity target the inhibition of FTO and ALKBH5, respectively (Su et al. 2020; Tang et al. 2021). Nonetheless, the efficacy of these drugs requires verification through in vivo studies.

Silencing of METTL3 can lead to the inactivation of NFκB, the reduction of proinflammatory cytokines, and the inhibition of extracellular matrix degradation. Therefore, METTL3 may be a potential target for the treatment of inflammatory diseases and conditions associated with tissue degradation (Liu, Zheng, et al. 2022). Interestingly, METTL3 has been suggested as a potential treatment target in osteoarthritis, whereas FTO has been suggested as a target in osteoporosis (Chen et al. 2021). The role of FTO in m6A variations among bone cells has been suggested to be a potential therapeutic marker to improve osteogenesis as a treatment for osteoporosis (Huang, Guo, Liu, et al. 2023). This approach might be promising for applications in periodontal regeneration.

Several m6A-related genes have been suggested as biomarkers as well as treatment options for inflammatory bowel disease (IBS) (Zhang et al. 2023). Substances such as resveratrol, curcumin, 2-hydroxy-4-methylthiobutyric acid, and butyrate have been shown to influence markers such as METTL3 and FTO, which further influence inflammatory genes and epithelial barrier structure to improve IBS. Similarly, findings have demonstrated the role of IGF2BP2 in the regulation of both inflammatory response and osteoclast differentiation in periodontitis (Ma et al. 2024). Host modulation strategies could be resourceful for patients in whom periodontal surgery is contraindicated, potentially further slowing periodontal breakdown.

The current knowledge of m6A function in various cells remains scarce, and a better understanding is essential prior to exploring therapeutical strategies. Most studies conducted to date remain experimental, and conclusions have been drawn from observations of preclinical animal models. Further research is needed to elucidate m6A-related signaling pathways and identify suitable targets for the treatment of inflammatory diseases such as periodontitis and peri-implantitis.

Ultimately, understanding patient-specific epigenetic profiles could lead to personalized treatment strategies. Epigenetic profiling of patients could help identify those who are more likely to respond to specific epigenetic therapies, thereby improving treatment outcomes and minimizing adverse effects. Such approaches may uncover novel biomarkers and therapeutic targets, facilitating the development of precision medicine strategies tailored to individual patient profiles and offering new alternatives for disease management and prevention.

## Conclusions

DNA and RNA methylation are tissue- and cell-specific regulatory mechanisms present in periodontal and peri-implant lesions and their associated enzymes suggested to contribute to disease. The microbiome and cellular composition, especially neutrophils and macrophage phenotype, can influence DNA and RNA methylation patterns. Further mechanistic and clinical investigations are essential to explore the signaling pathways of DNA and RNA methylation proteins, their diagnostic value, and their impact as therapeutic targets.

## Author Contributions

L. Larsson, C. Garaicoa-Pazmino, F. Asa’ad, contributed to conception and design, data analysis, drafted and critically revised the manuscript; P.M. Giraldo-Osorno, contributed to data analysis, critically revised the manuscript; W.V. Giannobile, contributed to conception and design, data analysis, critically revised the manuscript. All authors gave their final approval and agree to be accountable for all aspects of the work.

## Supplemental Material

sj-docx-1-jdr-10.1177_00220345241291533 – Supplemental material for DNA and RNA Methylation in Periodontal and Peri-implant Diseases Supplemental material, sj-docx-1-jdr-10.1177_00220345241291533 for DNA and RNA Methylation in Periodontal and Peri-implant Diseases by L. Larsson, P.M. Giraldo-Osorno, C. Garaicoa-Pazmino, W.V. Giannobile and F. Asa’ad in Journal of Dental Research
