# Supplementary material for: DNA and RNA Methylation in Periodontal and Peri-implant Diseases
Source: J Dent Res. 2024 Dec 4;104(2):131–9. doi: 10.1177/00220345241291533 (PMC11752639; doi:10.1177/00220345241291533)
Supplement: sj-docx-1-jdr-10.1177_00220345241291533 – Supplemental material for DNA and RNA Methylation in Periodontal and Peri-implant Diseases [file sj-docx-1-jdr-10.1177_00220345241291533.docx]

**Appendix**

**DNA and RNA methylation in periodontal and peri-implant diseases**

Lena Larsson, Paula Milena Giraldo-Osorno, Carlos Garaicoa-Pazmino, William V. Giannobile, Farah Asa’ad

**Table 1.** Characteristics and main findings of studies evaluating DNA methylation in periodontal/peri-implant diseases using in-vitro, ex-vivo, experimental and human models.

| ***Periodontal studies*** | | | | |
| --- | --- | --- | --- | --- |
| **Authors** | **Year** | **Study type** | **DNA methylation targets** | **Main findings** |
| de Oliveira et al. | 2011 | Ex-vivo | TLR2 and TLR4 | Major unmethylation of TLR4 gene promoter among healthy/periodontitis and non-smoking/smokers patients. Mosaic of methylated and unmethylated DNA was observed of the TLR2 gene promoter |
| Yin & Chung | 2011 | Ex-vivo | DNMT1 and H3K4me3 | Gingival epithelial cells from human biopsies exposed to *Porphyromonas gingivalis* and *Fusobacterium nucleatum* had decreased levels of DMNT1 and H3K4me3 expression. Pre-treatment with trichostatin A and sodium butyrate significantly enhanced gene expression of hBD2 and CCL20 in response to bacterial challenge. On the other hand, pre-treatment with DNMT inhibitor 5'-azacytidine increased hBD2 and CCL20 expression in response to *F. nucleatum*, but not to *P. gingivalis*. |
| de Faria Amormino et al. | 2013 | Ex-vivo | TLR2 | Positive correlation between periodontal inflammation/probing depths and DNA methylation. Hypermethylated profile and low gene expression of TLR2 within human periodontitis lesions |
| de Souza et al. | 2014 | Ex-vivo | Multiple | Mean methylation scores and frequency of methylated probes were significantly lower in genes related to the immune inflammatory transcription in biopsies from human periodontitis lesions |
| Miao et al. | 2014 | In-vitro | MMP-2 | Hypomethylation of MMP-2 promoter was consistent with constitutive pro-MMP-2 expression in PDL cells. *Treponema denticola*-mediated upregulation of MMP-2-related genes and chronic activation of pro-MMP-2 mimics key in vivo mechanisms of periodontal disease. |
| Benekanakere et al. | 2015 | Animal/In-vitro | TLR2 | DNA methylation of TLR2 within human gingival epithelial cells can modulate host innate defense mechanisms that may confer increased disease susceptibility to periodontitis as observed in both experimental and in-vitro studies |
| Larsson et al. | 2016 | Ex-vivo | 5mC, 5hmC, TET2 and DNMT1 | Larger proportion of TET2-positive cells was found in human periodontitis lesions compared to gingivitis. No differences between TET2 and IDH genes. Higher global levels of 5hmC in serum samples than in biopsies in patients with periodontitis. |
| Martins et al. | 2016 | Animal/In-vitro | DNMT1, acH3, TLR1, TLR2 and TLR4 | LPS exposure induces histone acetylation, downregulation of DNMT1 and activation of transcriptional coactivators (e.g., p300/CBP, NF-κB) and TLRs receptors (TLR1, TLR2 and TLR4) among human epithelial cells and experimental periodontitis. |
| Schulz et al. | 2016 | Ex-vivo | Multiple | Differential methylation patterns were observed among biopsies from patients with aggressive periodontitis, especially for CCL25 and IL-17C. CCL25 plays a role in T-cell development whereas IL-17C regulates innate epithelial immune responses |
| Asa'ad et al. | 2017 | Case control | COX-2 gene, TNF-α, IFN-γ and LINE-1 | Periodontal therapy resets DNA methylation of COX-2 gene in biopsies from human periodontal disease |
| Coêlho et al. | 2020 | Ex-vivo | MTHFR, DNMT3B, miR-9-1, miR-9-3, SOD-1 and CAT | DNMT3B polymorphism and methylated profile of miR-9-1 promoter region in human buccal mucosa cells were associated with periodontitis |
| Asa'ad et al. | 2023 | Cross-sectional | SNPs | Genetic analysis from saliva samples found associations between gene SNP/DNA methylation and periodontitis, tooth loss, low-grade inflammation and hyperglycemia |
|  |  |  |  |  |
| ***Peri-implant studies*** | | | | |
| **Authors** | **Year** | **Study type** | **DNA methylation targets** | **Main findings** |
| Daubert et al. | 2019 | Case control | 5mC | Increased levels of 5mC in PICF from human peri-implantitis lesions as compared to peri-implant health. DNA methylation may be affected by titanium dissolution products |
| Khouly et al. | 2022 | Ex-vivo | Multiple | Higher global DNA methylation levels were found in gingiva/peri-implant mucosa when compared to bone among periodontal and peri-implant samples. Similar global DNA methylation levels noted among sited with failed dental implants and periodontal health |

*TLR: Toll-like receptor; DNMT: DNA methyltransferase; H3K4me3: Histone H3 tri-methylated at Lys4; hBD2: Human β-defensin 2; CCL: CC chemokine ligand; MMP: Matrix metalloproteinase; PDL: Periodontal ligament; 5mC: 5-methylcytosine; 5hmC: 5-hydroxymethylcytosine; TET: Ten-eleven translocation; IDH: Isocitrate dehydrogenase; acH3: Acetylated histone H3; LPS: Lipopolysaccharide, CBP: CREB-binding protein; NF-κB: Nuclear factor kappa-light-chain-enhancer of activated B cells; IL: Interleukin; COX: Cyclooxygenase; TNF-α: Tumor necrosis factor-alpha; IFN-γ: Interferon-gamma; LINE: Long interspersed nuclear element; MTHFR: Methylenetetrahydofolate reductase; miR: MicroRNA; SOD: Superoxide dismutase; CAT: Catalase; SNP: Single nucleotide polymorphism; PICF: Peri-implant crevicular fluid.*

**Table 2.** Functional roles of m6A regulators in RNA metabolism (Modified from Jiang et al. 2021).

| **Type** | **m6A regulators** | **Biological function** |
| --- | --- | --- |
| Writers | METTL3 | Catalyzes m6A modification |
|  | METTL14 | Assists METTL3 to recognize the subtract |
|  | METTL16 | Catalyzes m6A modification |
|  | WTAP | Promotes METTL3-METTL14 heterodimer to the nuclear speckle |
|  | VIRMA | Guides the methyltransferase components to specific RNA region |
|  | RBM15 | Binds the m6A complex and recruit it to special RNA site |
|  | ZC3H13 | Bridges WTAP to the mRNA-binding factor Nito |
| Erasers | FTO | Removes m6A modification |
|  | ALKBH5 | Removes m6A modification |
| Readers | IGF2BP1 | Enhances mRNA stability |
|  | IGF2BP2 | Enhances mRNA stability |
|  | IGF2BP3 | Enhances mRNA stability |
|  | YTHDC1 | Promotes RNA splicing and translocation |
|  | YTHDC2 | Enhances the translation of target RNA |
|  | YTHDF1 | Promotes mRNA translation |
|  | YTHDF2 | Reduces mRNA stability |
|  | YTHDF3 | Mediates the translation or degradation |
|  | HuR | Unclear |

*m6A: N6-methyladenosine; METTL: Methyltransferase-like protein; WTAP: Wilms tumor 1-associated protein; VIRMA: Vir-like m6A associated protein; RBM: RNA binding motif protein; ZC3H: Zinc finger CCCH domain-containing protein; FTO: Fat mass and obesity associated protein; ALKBH5: Alkylation B homolog 5 RNA demethylase; IGF2BP: Insulin-like growth factor 2 mRNA-binding protein; YTHDC: YTH domain-containing protein; YTHDF: YTH N6-methyladenosine RNA binding protein; HuR: Human antigen R.*

**Table 3.** Characteristics and main findings of studies evaluating RNA modifications in periodontal/peri-implant diseases using in-vitro, ex-vivo, experimental and human models.

| ***Periodontal studies*** | | | | | |
| --- | --- | --- | --- | --- | --- |
| **Authors** | **Year** | **Study type** | | **m6A regulator** | **Main findings** |
| Lin et al. | 2020 | Ex-vivo | | Multiple | Genome-wide association data revealed dozen of m6A SNP may play a crucial role in the pathogenesis of human periodontal disease. Rs2723183 was predicted to regulate local gene IL-37 expression in periodontitis and change regulatory motif RXRA |
| Zhang et al. | 2021 | Ex-vivo | | Multiple | ALKBH5 and FMR1 are related to infiltrating monocyte abundance and affecting the expression of HLA-B and HLA-DOA in human periodontitis biopsies. Also, HuR and CBLL1 are significant regulators in immune reaction of TNF receptors/cytokines. |
| Li et al. | 2023 | Animal/in vitro | | FTO | TNF-α induced inflammatory response decreased the expression of FTO within cementoblasts in both in-vitro and experimental (apical periodontitis) models |
| Sun et al. | 2022 | Ex-vivo | | METTL3 | Lower expression of METTL3 among PDL cells exposed to static mechanical stress in periodontitis compared to periodontal health. METTL3 promoted osteogenic differentiation of PDL cells from periodontitis tissues |
| Huang et al. | 2023a | Animal/in vitro | | METTL3 and METTL14 | METTL3 and METTL14 regulate osteogenic differentiation and play indispensable roles in the regenerative potential of human PDL cells |
| Vignon et al. | 2023 | Ex-vivo | | Multiple | Some salivary ribonucleosides appear to be promising diagnostic biomarkers of periodontitis |
| Wang et al. | 2023 | Ex-vivo | | Multiple | Differentially methylated and expressed mRNAs were mainly involved in the regulation of stem cell differentiation, ossification, circadian rhythm and insulin secretion pathways. DNER and GNL2 are differentially m6A methylated in human tissues affected with periodontitis when compared to healthy tissues |
| Zhou et al. | 2023 | Animal/in vitro | | FTO | Advanced glycation end products impair bone marrow MSC osteogenesis in periodontitis/diabetes via FTO-mediated m6A modification of sclerostin |
| Chen et al. | 2024 | In vitro | | METTL3 | METTL3 regulates osteogenic differentiation of human PDL stem cells through LncRNA CUTALP |
| Huang et al. | 2024 | Animal/in vitro | | METTL3 and METTL14 | Regulates IL-6 expression via RNA m6A modification of Zinc transporter SLC39A9 and DNA methylation of IL-6 in PDL cells |
| Ma et al. | 2024 | Animal | | IGF2BP2 | IGF2BP2 responds to bacterial-induced stimuli and exhibits different expression patterns in early and advanced periodontitis lesions, and suggesting a dual role in immunomodulation and osteoclastogenesis during different stages of periodontitis |
| Sun et al. | 2024b | Ex-vivo/in-vitro | | METTL3 and IGF2BP1 | METTL3 promotes osteogenic differentiation of human PDL cells through IGF2BP1-mediated regulation of Runx2 stability |
| Zhang et al | 2024a | Ex-vivo/in-vitro | | Multiple | METTL3 promotes osteogenic differentiation of PDL stem cells from periodontitis patients by regulating the stability of LncRNA4114 |
| Zhang et al. | 2024b | Animal | | METTL3 | METTL3 promotes ribosome biogenesis and oxidative phosphorylation by activating Wnt/B-catenin/c-Myc signaling in LPS-treated osteoblasts and alleviated bone destruction in experimental periodontitis. |
|  |  |  | |  |  |
| ***Peri-implant studies*** | | | | | |
| **Authors** | **Year** | **Study type** | **m6A regulator** | | **Main findings** |
| Krishnamoorthy et al. | 2023 | Ex-vivo | METTL3 | | High expression of METTL3 might influence elevated levels of m6A RNA methylation in human peri-implantitis lesions. Dysregulation of m6A modification is risk factor associated with peri-implantitis |

*m6A: N6-methyladenosine; SNP: Single nucleotide polymorphism; IL: Interleukin; RXRA: Retinoid X receptor alpha; ALKBH5: Alkylation B homolog 5 RNA demethylase; FMR: Fragile X messenger ribonucleoprotein; HLA: Human leukocyte antigen; HLA-DOA: HLA class II histocompatibility antigen DO alpha chain; HuR: Human antigen R; CBLL: Casitas B-lineage lymphoma-transforming sequence-like protein; TNF: Tumor necrosis factor; FTO: Fat mass and obesity associated protein; METTL: Methyltransferase-like protein; PDL: Periodontal ligament; mRNA: messenger RNA; DNER: Delta and Notch-like epidermal growth factor-related receptor; GNL: Nucleolar GTP-binding protein; MSC: Mesenchymal stem cells; LncRNA: Long non-coding RNA; CUTALP: CutA Divalent Cation Tolerance-like Pseudogene; SLC39A9: Solute carrier family 39 member 9; IGF2BP: Insulin-like growth factor 2 mRNA-binding protein; Runx: Runt-related transcription factor; LPS: Lipopolysaccharide.*
